# Do Paramedics Have a Professional Obligation to Work During a Pandemic? A Qualitative Exploration of Community Member Expectations

**DOI:** 10.1017/dmp.2020.212

**Published:** 2020-06-24

**Authors:** Cameron Anderson, Julie Ann Pooley, Brennen Mills, Emma Anderson, Erin C. Smith

**Affiliations:** School of Medical and Health Sciences, Edith Cowan University, Joondalup, WA, Australia; School of Arts and Humanities, Edith Cowan University, Joondalup, WA, Australia

**Keywords:** mass casualty incidents, disaster medicine, emergency medical services, triage, public health surveillance

## Abstract

**Objectives:**

Previous research has identified a lack of clarification regarding paramedic professional obligation to work. Understanding community expectations of paramedics will provide some clarity around this issue. The objective of this research was to explore the expectations of a sample of Australian community members regarding the professional obligation of paramedics to respond during pandemics.

**Methods:**

The authors used qualitative methods to gather Australian community member perspectives immediately before the onset of the coronavirus disease 2019 (COVID-19) pandemic. Focus groups were used for data collection, and a thematic analysis was conducted.

**Results:**

The findings revealed 9 key themes: context of obligation (normal operations versus crisis situation), hierarchy of obligation (individual versus organizational obligation), risk acceptability, acceptable occupational risk (it’s part of the job), access to personal protective equipment, legal and ethical guidelines, education and training, safety, and acceptable limitations to obligation. The factors identified as being acceptable limitations to professional obligation are presented as further sub-themes: physical health, mental health, and competing personal obligations.

**Conclusions:**

The issue of professional obligation must be addressed by ambulance services as a matter of urgency, especially in light of the COVID-19 coronavirus pandemic. Further research is recommended to understand how community member expectations evolve during and after the COVID-19 coronavirus pandemic.

An epidemic of severe acute respiratory syndrome (SARS) coronavirus (SARS-CoV) affected 26 countries and resulted in more than 8000 confirmed cases in 2003.^1^ Most cases of human-to-human transmission occurred in the health-care setting in the absence of adequate infection control precautions. Implementation of appropriate infection control practices brought the global outbreak to an end, but not before it exposed the vulnerabilities of many health-care systems. Health-care professionals bore the brunt of the outbreak and were the most at-risk population for SARS, accounting for 21% of all cases worldwide and 45% of probable or suspect cases in Toronto, Canada, during the outbreak.^2^

Seventeen years later, another coronavirus (SARS-CoV-2) caused a worldwide pandemic. On January 30, 2020, the World Health Organization (WHO) declared the outbreak of SARS-CoV-2 to be a “Public Health Emergency of International Concern.” The disease caused by this outbreak was given the title “COVID-19” (coronavirus disease 2019) on February 11, 2020, and formally acknowledged as a pandemic on March 11, 2020—the first pandemic to be caused by a coronavirus.^3^ By April 2, the COVID-19 coronavirus pandemic passed 2 grim milestones: more than 1 million confirmed infections and 50,000 deaths worldwide.^4^

The impact on frontline health-care workers became evident once again. Figures from China’s National Health Commission indicated that more than 3300 health-care workers had been infected as of early March. In Italy, 20% of responding health-care workers were infected.^5^ Between February 12 and April 9, a total of 9282 (19%) of 315,531 COVID-19 cases reported to the Centers for Disease Control and Prevention (CDC) in the United States were health--care workers.^6^

Frontline health-care workers provide urgent care for patients with COVID-19, leading to stressful and exhausting work and are the most likely to be exposed to an infection. By April 15, hundreds of health-care workers around the world had died.^7^ As worldwide supplies of personal protective equipment (PPE) rapidly dwindled, frontline health-care professionals in every affected country had to carefully consider their situation. The difficult choice was between whether or not to provide care, or protect themselves. This is an ethical dilemma and is fundamentally challenging assumptions about professional obligation and personal risk.

When does the right to protect oneself from serious risk outweigh an obligation to respond to patients in need? There is no uncontroversial way to establish a threshold at which personal risk becomes an acceptable part of professional obligation to respond.^8^ Recent Australian research exploring these questions identified that paramedic decisions around professional obligation largely depend on their individual risk assessment, perception of risk, and personal value systems. The majority (86%) of paramedic participants favored the idea that professional obligation should not be considered an unlimited and absolute expectation.^9^ The expectations of community members regarding paramedic obligation to work are limited in the published literature,^10^ with no existing evidence establishing these expectations specifically in the context of pandemic response.

The objective of this research was to understand community member expectations regarding paramedic obligation to respond during a pandemic. As this research was conducted in the months leading up to the COVID-19 coronavirus pandemic, these findings provide a unique insight into how the community viewed the professional obligations of paramedics before a pandemic occurring.

## METHODS

This research applied a qualitative methodological design using focus groups. Recruitment of participants followed a multimodal strategy, including initial convenience and subsequent snowball sampling using a variety of social media platforms (including Facebook, Instagram, and LinkedIn). This nonprobability sampling technique is often used by qualitative researchers to recruit participants who are easily accessible and convenient to the researchers. The approach often includes the use of online social media resources that make participant recruitment convenient.

Qualitative data were collected from 41 participants (24 females and 17 males) across 4 focus groups conducted in Victoria, Australia, between October 2019 and February 2020. The participants were Australian community members aged 18 years or over. Anyone currently employed as a paramedic was excluded, although other health-care professionals were not.

A central set of questions and probes were developed by the research team. For consistency of approach, 2 research team members were present at all focus groups and 1 research team member facilitated all focus groups and interviews.

The focus groups ran for an average of 64 min. With the permission of participants, all focus groups were audio-recorded. Audio recordings were transcribed, and a thematic analysis was undertaken using the NVivo version 12 software package.

To analyze the data, a coding protocol was developed using a combination of several qualitative analytic approaches. This protocol included preliminary manual coding of the data to identify relevant themes.^11^ Additional coding was then used to identify any overarching themes represented in the data. For core coding categories, 2 independent members of the research team coded 20% of the data. Inter-coder agreement was assessed using the kappa coefficient, and agreement was high (0.85) for all coding. Discrepancies were resolved through discussion until 100% agreement on themes was achieved, and the remaining transcripts were divided between the 2 coders for independent coding.

Ethics approval was granted for this research by the Edith Cowan University Human Research Ethics Committee (Project Number: #20170).

## RESULTS

Thematic analysis revealed 9 key recurring themes: context of obligation (normal operations vs crisis situation), hierarchy of obligation (individual vs organizational obligation), risk acceptability; acceptable occupational risk (“it’s part of the job”), access to PPE, legal and ethical guidelines, education and training, safety, and acceptable limitations to obligation. The factors identified as being acceptable limitations to professional obligation are presented as further sub-themes: “physical health,” “mental health,” and “competing personal obligations.” These themes and sub-themes are reproduced in Table 1.

**TABLE 1:** Identified Themes and Sub-themes

Themes	Sub-themes
Context of obligation	Does the same professional obligation transcend normal operations into crisis situations?
Hierarchy of obligation	Obligations of individual paramedics versus organizational obligation to protect workers.
Risk acceptability	Determining level of risk acceptability; ramifications; managing risk that breaches a risk-acceptability threshold; controlled versus uncontrolled risk; timing of paramedic risk acceptability decisions.
Acceptable occupational risk	Expectation that a certain degree of risk is inherently “part of the job”.
Access to PPE	Paramedic professional obligation dependent on access to appropriate PPE.
Legal and ethical guidelines	Ethical versus legal obligation.
Education and training	Expectation that with specialized education comes obligation to respond.
Safety	Acceptability of physical risk versus health risk.
Acceptable limits to obligation	Physical health; mental health; competing personal obligations.

## DISCUSSION

The themes and sub-themes identified during the analysis of the focus group data provided valuable insights, which are discussed below.

### Context of Obligation (Normal Operations vs Crisis Situation)

When asked if paramedics have an obligation to respond during a pandemic, participants were emphatic that a “degree” of obligation exists. However, much discussion focused on whether the same professional obligation transcended normal operations into crisis situations. Participants found it difficult to articulate clearly whether they believed paramedics retained a professional obligation to respond during pandemics, or whether leeway should be provided given the extreme personal risk associated with the conditions they are being asked to work under.

This finding supports the need for *crisis standards of care*, defined as a substantial change in usual health-care operations and the level of care it is possible to deliver, which is made necessary by a pervasive (eg, pandemic) or catastrophic (eg, earthquake) disaster.^12,13^ This change in the level of care delivered is justified by specific circumstances and should be formally declared by government. The formal declaration that crisis standards of care are in operation enables specific legal and regulatory powers and protections for health-care providers, particularly around obligation to work in the face of risk and in the necessary tasks of allocating and using scarce medical resources and implementing alternate care facility operations. Importantly, the community should be informed as to what they can expect from their health-care providers when these standards are in effect, and what circumstances might trigger such a declaration.

There was consensus that an underlying principle of *duty of care* guided paramedic obligation to respond during a pandemic, however, that principle was most commonly linked to normal day-to-day operations and not necessarily during crisis situations like a pandemic. There was considerable discussion around whether an obligation to respond should extend to crisis situations where the point of care may transfer from an individual patient to the greater population. In line with discussion around crisis standards of care, it was largely agreed across all focus groups that when the point of care transfers to the general population that a new standard of acceptability for professional obligation needs to be determined. This finding supports previous research that identified confusion among community participants regarding the extent of paramedic obligation and how it changed between normal day-to-day operations into the crisis situation context.^9^

One participant conveyed this sentiment:

“See I think they [paramedics] have an obligation to the community. They apply to be a paramedic and go through training and then they choose that career path because they want to contribute to their community, so yes, I think that means they have a duty to the community. But I am not sure you can put clear parameters around that duty. Like when does it become too much to expect from them?”

Another participant added:

“I think the fact that they have an obvious obligation to respond during a normal day, with individual patients, is pretty uncontroversial. But it’s much more difficult to say what their obligation is during a pandemic. That’s not a normal situation, so normal rules probably don’t apply.”

### Hierarchy of Obligation (Individual vs Organizational Obligation)

Further discussion attempted to clarify some type of hierarchy or scaffolding for professional obligation. This largely focused on the notion that obligation exists at 2 levels: the *individual paramedic* and the *organization.* There was a general consensus that while paramedics have an individual degree of obligation to respond during a pandemic, the ambulance services have a much higher level of organizational obligation to protect the health and well-being of the paramedic workforce. This organizational obligation needs to be fulfilled before any individual degree of obligation can be expected.

“The services need to provide clear guidance around obligation. They can’t just say it comes down to not expecting paramedics to put themselves at risk. How are they [individual paramedics] meant to determine how much risk is acceptable?”

Another participant picked up on this line of thinking:

“Yes, how can you base [their obligation to work] on acceptable risk, but then not clearly define how much risk is actually “acceptable”? Can they go on their gut feeling, you know, intuition, or do they have to follow pre-set measurements?”

### Risk Acceptability

Clarifying how much risk is “acceptable” regarding paramedic obligation to respond during a pandemic dominated much of the conversation across the focus groups. Two key sub-themes consistently emerged: What level of risk is acceptable? And; what happens if a paramedic believes that level of acceptability has been breached? The point at which risk becomes potentially “life threatening” was raised in all focus groups as being 1 threshold for unacceptability:

“I think they have a duty of care, but I don’t think they should risk their lives.”

“You should be able to have an expectation when you go off to work that you’re going to go home.”

Discussion of risk acceptability often evolved into consideration of what would happen should a paramedic perceive a risk to breach the acceptability threshold. This identified an additional sub-theme: controlled versus uncontrolled risk. There was general consensus among participants that being a paramedic should inherently come with an understanding that the job will expose them to a certain level of controlled risk. Where the discussion diverted into differing opinions was around the acceptability of controlled risk versus uncontrolled risk—with uncontrolled risk (ie, risk of exposure to infectious disease during a pandemic), on the whole, being deemed less acceptable than controlled risk:

“Controlled risk is one thing, sure, they have to expect an element of that as part of their job, but surely it becomes something different when managing that risk is out of their hands, such as during a pandemic, it’s that uncontrolled risk that is pretty unacceptable, such as taking it home to family.”

Another sub-theme to emerge within the discussion on acceptability of risk was the timing associated with paramedic decisions regarding risk acceptability. It was largely believed that paramedics had the right to determine whether or not they actually turned up for a shift, but once they had agreed to work they had also accepted the subsequent risks that came with that shift, including responding to potential or confirmed cases during a pandemic.

“If they make the decision not to work, then fine, that’s one thing. But if they show up at the beginning of the shift, then they have to take what comes. They shouldn’t be able to pick and choose what jobs, or patients they agree to see”.

### Acceptable Occupational Risk (“It’s Part of the Job”)

There was general consensus among participants that paramedics will play an integral frontline role in responding to pandemics. Further discussion focused on how this frontline role could result in paramedics being exposed to infectious disease and whether some level of exposure should be considered an inherent “part of the job”.

“Listen, if you sign up for this, you do the training, and you still want to be out there as a paramedic, then I think you have to just accept there will be certain types of risks associated with that, [like] being exposed to infectious disease.”

Discussion of this theme invariably segued into subsequent conversation regarding access to personal protective equipment (PPE) in all focus groups. There were clear linkages between discussions of risk being “part of the job” with subsequent expectations that paramedics would have some appropriate level of protection against those risks.

### Access to PPE

The provision of PPE was overwhelmingly identified as being associated with a professional obligation to respond. If PPE is provided, then response is expected. If PPE is lacking, then it comes back to questions around acceptable levels of risk and what that threshold will be for individual paramedics.

Access to PPE has certainly been an issue globally during the COVID-19 coronavirus pandemic.^14,15^ Australia’s health-care workforce has been traumatized by the limited access to protective equipment in the fight against coronavirus. A survey of 245 Australian doctors in April 2020 identified that 61% felt pressure from other staff not to wear a mask and more than half felt guilt or shame for wearing one. A further 86% reported feeling anxious about the level of PPE provided to them during the pandemic, and 83% did not trust that the Australian guidelines were adequate. This survey also highlighted that many doctors were being threatened and warned against wearing PPE by their employers.^16^

It is likely that paramedics on the frontlines will hold similar concerns as well. And these concerns, it appears, are valid according to many of the community participants in this research. One participant offered the following reflection on the topic:

“If they have PPE, then yes, I expect them to be responding in a pandemic, but if not, it’s a hard one. Because we still need them to be out there in the community helping people, but not if it means they are at high risk of getting sick themselves, and then taking that home to their own families.”

Another participant considered the responsibility of the ambulance services to the paramedics themselves:

“Is it even reasonable to send them in if we can’t protect them?”

It was also interesting to note a sub-theme emerging of the community wanting to protect the paramedics during a pandemic:

“I think if I was the patient or just a member of the community and I’d exposed a paramedic and his or her family to that, I don’t think I could forgive myself either.”

But it appears that this concern is not without limits. Overwhelmingly, participants did not expect paramedics to put themselves in harm’s way or work if they weren’t comfortable—unless that meant that they or their families would not get timely access to emergency medical services during a pandemic.

“Well of course, I wouldn’t want them to get sick, but at the end of the day it’s the job they have chosen. And if I need them during a pandemic, and they have the training and the PPE to protect them, then yes, I expect them to show up.”

This sentiment was shared by other participants:

“Yeah, I still want them to be there if I need them, even if showing up puts them at some level of risk.”

“Yes, they still have a job to do, they signed up for this. If I need them in a pandemic, I am expecting that they show up at my door.”

### Legal and Ethical Issues

The legal and ethical dilemmas faced by frontline health professionals during disaster response were highlighted by the experience of Memorial Medical Centre in New Orleans during the evacuation of patients after Hurricane Katrina and the subsequent flooding. Criminal charges were filed against a doctor and 2 emergency nurses for failure to meet standards of care. Questions about what may lead to censure, penalties from licensing boards, or lawsuits were subsequently asked by many health professionals, and led to joint publications and commentary by major health professional groups in the United States.^17^

The legal and ethical dilemmas faced by paramedics were raised in all focus groups, with a general consensus that there is no clear understanding as to whether paramedics have a legal or ethical obligation to respond. Furthermore, there was also a lack of clear understanding regarding what the ramifications would or should be for failing to meet that duty in the face of considerable personal risk.

One participant summarized the confusion:

“But what do we even mean by this issue of obligation, or duty of care, what does it even mean, is it legal? Is it ethical?”

The Australian National Registration and Accreditation Scheme for health professions (NRAS) was established in 2010 to ensure the safety of consumers of health services by registering health practitioners. The Australian Health Practitioner Regulation Agency (AHPRA) is the national organization responsible for implementing the National Registration and Accreditation Scheme (the National Scheme) across Australia. Paramedics in Australia are now considered health-care professionals under this scheme and as such, issues around regulation and any associated code of ethics for prehospital response should be developed as a priority. Professional associations and codes of ethics can play an important role in helping to articulate the fundamental professional obligations of paramedics.

Educators can provide paramedic students with an understanding and appreciation of these fundamental responsibilities by focusing attention on both the medical and ethical challenges and consequences involved with disaster response. To help experienced paramedics make these choices, ambulance services must provide their employees with the best current information about risks, aiding paramedics to make defensible decisions in difficult circumstances.

Coming to a more comprehensive understanding of the legal issues, as well as the ethical issues and social expectations in advance of a pandemic, would assist paramedics to respond willingly and appropriately. The urgency of this has been clearly demonstrated with the emergence of the COVID-19 coronavirus in early 2020 following the completion of these focus group discussions. The reality of the pandemic has already resulted in mass global shortages of PPE and reports of health-care workers around the globe starting to weigh up their professional obligations with personal risk. This is where clearly articulated guidance around what that obligation was would be very beneficial.

### Education and Training

Education and training were often raised as factors influencing professional obligation. There was a general expectation that, with the high level of education provided to paramedics comes a certain level of obligation to “pay that forward” to the community in times of crisis. However, education of infectious disease and the use of infection control systems does not ensure that health-care professionals comply with standard practices, and infections still regularly occur in the health-care workplace.^17^ A factor that complicates the issue of paramedic compliance with standard practices is the environment, with the unpredictable, uncontrolled, and suboptimal conditions encountered by paramedics, limiting the opportunity to apply adequate infection control procedures.

### Safety

Thematic analysis identified the concept of safety as a recurring theme. Of interest, the participants overwhelmingly agreed that paramedics should be able to expect a safe working environment when it came to the threat of *physical harm* or violence. However, the same absolute expectation of safety from *health harm* was not unanimously supported. Some risk of infectious disease exposure was seen to be an inherent part of the paramedic role.

“It’s part of their job, I mean, they have trained for it, they have the right protective gear, then yes, they should expect to have to deal with infectious disease.”

“For sure, I think they have a right to expect to be safe from physical violence, but I don’t necessarily think they have a right to expect to be safe from health perspective. I mean, attending patients with diseases is surely just part of their job that they have to accept?”

### Acceptable Limitations

Research participants were asked to consider what would be acceptable reasons for limiting professional obligation to respond during a pandemic. Responses identified 3 key themes: physical health, mental health, and competing personal obligations. Within each of these themes, participants provided specific examples of when these limits could be applied. These individual examples were further analyzed to identify recurring sub-themes. For example, within the key theme of physical health, many participants suggested that paramedics with existing illnesses, such as asthma, diabetes, and heart disease, could have their responsibility limited when these illnesses could impact on the paramedic’s ability to fulfill their role. There was recognition that a such limitations of responsibility necessary increase the workload and associated risk for other responders not fitting into 1 of these categories.

These responses were grouped together as 1 sub-theme: pre-existing chronic illness that may inhibit effective response. The key themes and sub-themes are presented in Table 2.

**TABLE 2:** Examples of Acceptable Limitations on Professional Responsibility

Physical Health Issues	Mental Health Issues	Competing Obligations
Pregnant females; pre-existing chronic illness; paramedics who become infected during the pandemic	Pre-existing mental health issues; mental health issues that develop during course of pandemic (eg, posttraumatic stress disorder)	Single parents; parents of children with chronic illness; both parents are health-care workers

The themes and sub-themes that emerged support similar findings from previous Australian research that also identified that physical health, mental health, competing personal obligations and notions of risk acceptability could all be used to guide the development of clear guidelines around acceptable limitations on paramedic professional responsibility.^9^

### Limitations

The findings reported in this publication are subject to several limitations. From a methodological perspective, the sampling methods used are an example of nonprobability sampling, rendering it impossible to determine how representative these findings are of the broader population. That being said, researchers often use nonprobability samples for projects that are qualitative in nature, where the researcher’s goal is in-depth, contextual understanding rather than more general, nominal understanding. The results are based on the responses of a small number of community members (*n* = 41) from Australia. Future research should repeat this methodology and introduce other methodologies with a broader range of community members.

The qualitative methodologies used throughout this research allow for exploration of individual perceptions, feelings, and needs. They are not, however, without their limitations. For example, descriptions of responsibility and risk by individual research participants will potentially be biased by their reliability of recall, previous experience and the way in which the discussion is framed. Furthermore, an individual’s intrinsic psychological processes may also influence how they experience certain types of events. Notwithstanding these methodological limitations, this study has provided an important contribution of new knowledge to help evolve the existing evidence-base on paramedic role and responsibility, particularly surrounding response during a pandemic.

## CONCLUSIONS

This research provides an important and unique insight into the expectations of community members regarding paramedic obligation to respond during pandemics. The findings support previous research that highlights a lack of clarity regarding the concept of professional obligation, specifically, where does it begin and end? This must be addressed as a matter of urgency through the development of clear guidelines outlining obligation to respond under both normal day-to-day operations and during crisis situations—where crisis standards of care are needed. This is necessary to ensure community members understand when (and when not) to call an ambulance and how ambulance availability may be impacted when pandemics place health systems under duress.

This research highlights a strong belief that paramedics are entitled to a work environment free from threat of physical harm, and that paramedics may refuse to enter an incident scene they deem unsafe. However, this believe is challenged when the risk of infectious disease is introduced. Participants had an expectation that an ambulance would be available to them when and where required, and that the ambulance service would take care of any safety implications arising from the infectious disease risk.

This research has provided additional insight into community member expectations regarding acceptable limitations on professional obligations for paramedics during a pandemic. However, it will be important to see how these expectations evolve now that the participants will have real-world lived experience of a pandemic. Additionally, it will be critical to validate these results using a quantitative methodology across a broad sample size to give a more robust picture of the community’s expectations of their paramedics during a pandemic.
